# Comparison of speed versus complexity effects on the hemodynamic response of the trail making test in block designs

**DOI:** 10.1117/1.NPh.5.4.045007

**Published:** 2018-12-08

**Authors:** David Rosenbaum, Leonore Blum, Paul Schweizer, Andreas J. Fallgatter, Martin J. Herrmann, Ann-Christine Ehlis, Florian G. Metzger

**Affiliations:** a University Hospital Tuebingen, Department of Psychiatry and Psychotherapy, Tuebingen, Germany; b University of Tuebingen, Center of Integrative Neuroscience, Cluster of Excellence, Tuebingen, Germany; c University of Tuebingen, LEAD Graduate School and Research Network, Tuebingen, Germany; d University Hospital Wuerzburg, Department of Psychiatry, Psychosomatics and Psychotherapy, Center of Mental Health, Wuerzburg, Germany; e University Hospital Tuebingen, Geriatric Center, Tuebingen, Germany

**Keywords:** functional near-infrared spectroscopy, trail making test, processing speed, task complexity

## Abstract

The use of functional near-infrared spectroscopy (fNIRS) in block designs provides measures of cortical activity in ecologically valid environments. However, in some cases, the use of block designs may be problematic when data are not corrected for performance in a time-restricted block. We sought to investigate the effects of task complexity and processing speed on hemodynamic responses in an fNIRS block design. To differentiate the effects of task complexity and processing speed, 20 subjects completed the trail making test (TMT) in two versions (TMT-A versus TMT-B) and three different speed levels (slow versus moderate versus fast). During TMT-A, subjects are asked to connect encircled numbers in numerically ascending order (1-2-3…). In the more complex TMT-B, subjects are instructed to connect encircled numbers and letters in alternating ascending order (1-A-2-B…). To illustrate the obscuring effects of processing speed on task complexity, we perform two different analyses. First, we analyze the classical measures of oxygenated blood, and second, we analyze the measures corrected for the number of processed items. Our results show large effects for processing speed within the bilateral inferior frontal gyrus, left dorsolateral prefrontal cortex, and superior parietal lobule (SPL). The TMT contrast did not show significant effects with classical measures, although trends are observed for higher activation during TMT-B. When corrected for processed items, higher activity for TMT-B in comparison to TMT-A is found within the SPL. The results are discussed in light of recent research designs, and simple to use correction methods are suggested.

## 1 Introduction

Functional near-infrared spectroscopy (fNIRS) is an optical imaging method that is based on the physical properties of light in the near-infrared (NIR)-spectrum. Specifically, the method is based on the principle that light in the NIR-spectrum is capable of penetrating biological tissues, such as skin and skull, and is absorbed to different degrees depending on thickness, density, and optical properties of the tissue. Due to these properties, it is possible to measure relative changes in oxygenated ($O_{2}\mathrm{Hb}$) and deoxygenated (HHb) hemoglobin in the human brain by placing fNIRS-sender and receiver optodes on a subject’s head. The penetration depth of the NIR-light is about 2 to 3 cm.^1^^,^^2^ Compared with other imaging methods, fNIRS has some important advantages.^3^ The method is relatively economic, comparably easy to use, relatively insensitive to movement artifacts, has a high time resolution, and can be used in mobile applications. Therefore, fNIRS has been used extensively in environments and subject populations, where other neuroimaging methods could not be implemented. For example, using fNIRS, it is possible to measure cortical activation while subjects perform job interviews in ecologically valid environments as in the Trier social stress test^4^^,^^5^ or while they perform cognitive tasks, such as the verbal fluency test (VFT)^6^^,^^7^ or the trail making test (TMT).^8^^–^^14^

While some of these tasks can be implemented within an event-related design, some are typically used with block designs. Importantly, using block designs in many cases preserves the ecological validity of a given task. For example, when subjects perform a free speech, it is ecologically valid to let them do so for the duration of a whole block instead of demanding single (and often simple) reactions to certain events. Usually, within a block design, the task and respective control conditions are performed for a certain duration of time (e.g., 30 to 40 s), and hemodynamic responses are averaged over this time period. In this way, fNIRS has been used to assess cortical hemodynamic changes during the performance of different versions of the TMT. The TMT is a neuropsychological test with two parts: TMT-A and TMT-B. During TMT-A, subjects are asked to connect numbers written in circles in ascending numerical order (1-2-3…) while during TMT-B, subjects are asked to connect alternating encircled numbers and letters in numerical and alphabetical order (1-A-2-B…). The TMT-A assesses visuospatial abilities and the speed of information processing, whereas the TMT-B additionally assesses the executive function of task switching. As the TMT-A is the easier task, it could be assumed that hemodynamic responses are higher during TMT-B—the more demanding task—in brain areas that are recruited by both tasks. However, past investigations on the subject did not always find this effect, especially when time-restricted versions were used.^8^^,^^9^

One explanation why differences between TMT-A and TMT-B are not always observed may lie within different factors that contribute to the hemodynamic response: speed and task complexity. From the perspective of a computational model, we assume that during a task block the hemodynamic response of a given subject is positively related to the number of finished items (processing speed) that have been performed. On the other hand, tasks that are more complex should recruit more brain areas as more cognitive functions are needed for task completion and—when controlled for processing speed—should result in higher hemodynamic responses within a given subject. Usually, during TMT-A, more items are finished than during TMT-B, but the latter is a far more complex task, so both effects (complexity versus processing speed) might cancel each other out in many regions of interest (ROIs). This also becomes plausible when considering typical experimental comparisons: when the TMT-A is used as a comparison condition for TMT-B, both tasks must be equal in their attributes except for the variable of interest. As during the TMT-A more items are processed than during TMT-B, a correction method needs to be implemented. Such a correction method—e.g., by dividing the activation during the block by the number of completed items—would result in a parameter that reflects the relative blood oxygenation per item, comparable to the average blood oxygenation during an event-related design, in which hemodynamic responses are averaged over each processed item.

Evidence from the effects of task complexity and processed items also comes from other research areas. For example, in a recent study by Artemenko et al.,^15^ higher activity within areas of the cognitive control network was observed during the computation of less complex mathematical equations in comparison to more complex mathematical equations. This inverse association of complexity and hemodynamic responses was no longer significant when “performed computation” was used as a covariate.^15^ However, with respect to complexity, more complex mathematical operations—such as the carry-over effect in mathematical operations—are known to induce higher frontal cortical activation than less demanding mathematical operations. This effect is considered to represent increased working memory load during carry-over computations.^16^ In line with this, hemodynamic responses are also positively associated with working memory load in n-back tasks.^17^^,^^18^

To test the effects of task complexity and processing speed, we investigated the hemodynamic responses during a block design of the TMT-A and TMT-B in the frontal and parietal cortex in three different speed conditions: slow speed, medium speed, and fast speed. In detail, we investigated blood oxygenation in the dorsolateral prefrontal cortex (DLPFC), inferior frontal gyrus (IFG), and superior parietal lobule (SPL)/somatosensory association cortex (SAC). We assumed to observe an increase in $O_{2}\mathrm{Hb}$ levels from slow- to fast-speed conditions and higher activity in the TMT-B than the TMT-A when controlled for processing speed. This hypothesis is based on two assumptions: the hemodynamic response within a certain subject within a block design is positively associated to the number of items being processed (first assumption) and the complexity of the task (second assumption).

## 2 Material and Methods

### 2.1 Participants

Twenty healthy subjects were recruited for this study. The ethics committee at the University Hospital and University of Tübingen approved this study. Further, all subjects gave written informed consent. Exclusion criteria were acute mental or physical illness, neurological disorders, and chronic or acute diseases that affect brain functioning, such as diabetes or kidney failure. Out of the 20 subjects, 12 participants were female; the average age was 27 years (SD=6.21), with 17.9 (SD=4.43) years of education. All subjects were right-handed.

### 2.2 Procedures

During the measurement, subjects sat on a comfortable chair with a wooden clipboard in front of them. The clipboard was fixated on the table with a steepness of ∼70  deg to allow working on a sheet of paper in upright head position and without horizontal head movements to minimize pressure on the optode fibers and resulting movement artifacts. Before the measurement, subjects completed a questionnaire assessing their demographic data and received instructions for the experiment. Afterward, they completed a version of the TMT-A and TMT-B in a slow (TMT-A) and fast manner (TMT-B) to become familiar with the tests and different processing speeds. In this training phase, subjects were given as much time as they needed to complete the whole test. Each version of the TMT consisted of 40 items. Subjects were instructed to work in the slow condition slowly, but not in a manner that they slow down with effort (e.g., by performing in very slow motion), in the fast condition as fast as possible, and in the moderate condition in between the speed of the slow and fast condition. At the beginning of the experimental blocks, subjects were asked to close their eyes and a pencil was placed in their hand. Afterward, subjects were instructed about the condition that would follow (TMT-A/TMT-B in slow/moderate/fast speed) and a corresponding test form was placed on the clipboard. Then, subjects were asked to open their eyes and start immediately with a 25-s block of task performance followed by 30-s rest. Afterward, the subjects were asked to close their eyes and the instruction for the next condition was given. In total, 18 blocks were assessed with three repetitions of each condition (TMT-A versus TMT-B in the conditions slow versus moderate versus fast) in a randomized order.

### 2.3 Functional Near-Infrared Spectroscopy

We used a continuous wave, multichannel near-infrared spectroscopy (NIRS) system (ETG-4000 Optical Topography System; Hitachi Medical Co., Japan) with a temporal resolution of 10 Hz. Data were recorded with a semiconductor laser and avalanche diodes at two wavelengths (695±20 and 830±20  nm) with 4.0±0.2  mW for each wavelength at each optode. In this study, we used three probesets placed on an electrode cap: 2 frontal probesets (reference points F3 and F4 according to the international 10–20 system^19^) with 9 optodes each and one parietal probeset (reference point Pz) with 15 optodes. The electrode caps were positioned to 10–20 system reference points Fpz and Cz for each subject to guarantee correct optode placement. The whole setup consisted of 46 channels, covering parts of the bilateral DLPFC, IFG, and the SAC. For a detailed description of the probesets, see Rosenbaum et al.^5^ Corresponding brain areas of each channel were extrapolated from reference points as in the work by Singh et al.^20^ and Tsuzuki et al.^21^^,^^22^ based on the Colin 27 template.

Raw data were exported as TBL directory from the NIRS machine and reconstructed with self-written MATLAB code. The changes in absorbed NIR-light were transformed into relative $O_{2}\mathrm{Hb}$ and HHb levels by means of a modified Beer–Lambert law. fNIRS preprocessing was performed with MATLAB R2017a (MathWorks Inc., Natick) and included the following steps: bandpass filtering (0.001 to 0.1 Hz) based on discrete cosine transform (DCT)-II and inverse DCT-II filters, correlation-based signal improvement according to,^23^ interpolation of single high artifact-loaded channels by visual inspection, independent component analysis (ICA) based reduction of clenching artifacts, and a further low cutoff filtering at 0.01 Hz. Note that the TMT in some cases induces high arousal artifacts that cannot be corrected by the ICA procedure due to too frequent high-amplitude signals. In this dataset, eight subjects showed such artifacts. In these cases, the signal was corrected by a principal component analysis (PCA) reduction of the first component.^24^ Additionally, data of all subjects were corrected for global signal changes by a Gaussian PCA-based kernel filter.^25^ Finally, we standardized each subject’s fNIRS data by the standard deviation of the concatenated signal of all channels. The 25-s blocks for each condition were averaged with a 10-s baseline correction and a linear detrending. Data were analyzed for five ROIs: SPL, bilateral DLPFC, and IFG chosen based on previous fMRI and NIRS studies on the subject.^8^^,^^11^^,^^26^ Brain maps were computed with self-written MATLAB routines. The MATLAB code of the analysis is available on request.

### 2.4 Data Analysis

Statistical analysis was performed with IBM SPSS Statistics Version 24. For behavioral (performance) and fNIRS data, repeated measurement analyses of variances (ANOVAs) with the within-subject factors complexity (TMT-A versus TMT-B) and speed (slow versus moderate versus fast) were performed. fNIRS data were analyzed separately for each of the five ROIs, with correction for multiple testing of post-hoc analysis by the procedure of Armitage-Parmar.^27^ As post-hoc test, we used planned t-tests and linear contrast. We assumed to find an increase from slower to faster processing and higher hemodynamic responses during TMT-B in comparison to TMT-A. For effect sizes, Cohen’s d and partial $\eta^{2}$ is reported. As we assumed that a correction for the number of performed computations would be needed to bring out the effects of task complexity, we performed two secondary analyses of the fNIRS data with corrected $O_{2}\mathrm{Hb}$ measures. To correct for performed computations, we used two different approaches. First, we regressed the average number of completed items per condition out of the hemodynamic response for each subject individually over all conditions and further analyzed residuals. Further, as the within-subject regression approach is not suitable for every NIRS investigation, we used an alternative more simple approach, in which we computed the ratio of a subject’s given $O_{2}\mathrm{Hb}$ concentration and the average performance in the corresponding condition, resulting in the measure “$O_{2}\mathrm{Hb}$ per solved item” [(mmol×mm)/item]. In this way, the $O_{2}\mathrm{Hb}$ values were set in relation to the individual number of processed items resulting in a metric comparable to the analysis of an event-related design, namely the relative blood oxygenation per solved item (during the block). Note that errors have not been used as covariates as error rates were very low (on average, below one error in five assessed blocks).

## 3 Results

### 3.1 Behavioral Data

With respect to behavioral performance in terms of completed items, a two (TMT) by three (speed) repeated measurement ANOVA showed significant main effects for TMT [$F_{\left(1,19\right)}=23.19$, p<0.001, $\eta^{2}=0.55$] and processing speed [$F_{\left(2,38\right)}=93.41$, p<0.001, $\eta^{2}=0.83$] as well as an interaction between both variables [$F_{\left(2,38\right)}=34.84$, p<0.001, $\eta^{2}=0.67$]. Not surprisingly, the main effects indicated more processed items during TMT-A than TMT-B and a linear increase in processed items from slow- to fast-processing conditions. However, the interaction of TMT by processing speed indicated that the differences between TMT-A and TMT-B were higher in the fast-processing condition than in moderate and slow conditions (see Table 1). Indeed, post-hoc analysis of paired t-tests revealed only significant differences between TMT-A and TMT-B in the fast condition [$t_{\left(19\right)}=7.49$, p<0.001, d=1.68]. Correspondingly, with respect to speed, highest effect sizes were observed for TMT-A between moderate- and fast-speed conditions [TMT-A: $t_{\left(19\right)}=10.63$, p<0.001, d=2.38; TMT-B: $t_{\left(19\right)}=5.36$, p<0.001, d=1.2), followed by increases from slow to moderate speed [TMT-A: $t_{\left(19\right)}=6.17$, p<0.001, d=1.38; TMT-B: $t_{\left(19\right)}=6.5$, p<0.001, d=1.45].

**Table 1:** Number of processed items and errors during TMT-A and TMT-B in the three speed conditions.

	TMT-A		TMT-B	
Variable	Mean	SD	Mean	SD
Processed items—slow	13.9	4.6	13.5	3.4
Processed items—moderate	19.2	5.7	17.8	5.2
Processed items—fast	30.1	5.8	22.5	5.8
Errors—slow	0.02	0.07	0.05	0.12
Errors—moderate	0.03	0.10	0.10	0.2
Errors—fast	0.20	0.31	0.23	0.27

With respect to error rates, we found a main effect for speed [$F_{\left(2,38\right)}=10.46$, p<0.001, $\eta^{2}=0.35$], which was driven by an increase in error rates from moderate- to fast-processing speed [$F_{\left(1,19\right)}=9.11$, p<0.01, $\eta^{2}=0.32$]. However, average error rates were very low overall (below one per condition).

### 3.2 Functional Near-Infrared Spectroscopy Data (mmol×mm)

As a first descriptive analysis, we checked the (uncorrected) activation of each channel against a zero mean distribution for each condition in multiple t-tests. Significant activations were found in each condition (see Table 2).

**Table 2:** Significant channels of the ROIs tested against zero in the experimental conditions. Note that p-values are not corrected for multiple tests, since the testing is used for descriptive purposes.

		TMT-A slow		TMT-B slow		TMT-A moderate		TMT-B moderate		TMT-A fast		TMT-B fast	
ROI	Channel	t	p-value	t	p-value	t	p-value	t	p-value	t	p-value	t	p-value
Left IFG	6	—	—	—	—	3.08	**0.006**	3.67	**0.002**	3.34	**0.003**	4.20	**0.000**
Left IFG	7	—	—	—	—	—	—	—	—	2.29	**0.034**	—	—
Left IFG	8	—	—	—	—	—	—	2.42	**0.026**	3.11	**0.006**	3.52	**0.002**
Left IFG	9	—	—	—	—	—	—	—	—	—	—	—	—
Left DLPFC	10	—	—	—	—	—	—	—	—	—	—	—	—
Left DLPFC	11	—	—	—	—	—	—	—	—	2.19	**0.041**	—	—
Left DLPFC	12	—	—	—	—	—	—	—	—	—	—	—	—
Right IFG	18	—	—	—	—	—	—	—	—	—	—	—	—
Right IFG	19	—	—	—	—	—	—	—	—	2.43	**0.025**	2.45	**0.024**
Right IFG	21	—	—	—	—	—	—	—	—	—	—	—	—
Right IFG	22	—	—	2.14	**0.045**	—	—	2.22	**0.039**	3.78	**0.001**	4.39	**0.000**
Right DLPFC	20	—	—	—	—	—	—	—	—	2.53	**0.020**	—	—
Right DLPFC	23	—	—	—	—	2.96	**0.008**	2.95	**0.008**	3.77	**0.001**	2.13	**0.046**
Right DLPFC	24	—	—	—	—	—	—	—	—	—	—	2.28	**0.034**
SAC	25	2.20	**0.040**	3.28	**0.004**	6.26	**0.000**	3.65	**0.002**	4.06	**0.001**	5.80	**0.000**
SAC	26	—	—	2.57	**0.019**	2.43	**0.025**	2.77	**0.012**	3.65	**0.002**	5.02	**0.000**
SAC	27	—	—	2.16	**0.043**	—	—	2.48	**0.023**	3.38	**0.003**	3.80	**0.001**
SAC	28	2.33	**0.031**	3.18	**0.005**	5.36	**0.000**	3.40	**0.003**	3.70	**0.002**	4.55	**0.000**
SAC	30	—	—	3.52	**0.002**	4.24	**0.000**	3.96	**0.001**	4.13	**0.001**	4.91	**0.000**
SAC	31	—	—	—	—	—	—	—	—	—	—	2.41	**0.026**
SAC	32	3.26	**0.004**	5.06	**0.000**	5.25	**0.000**	6.30	**0.000**	4.45	**0.000**	4.83	**0.000**
SAC	35	—	—	—	—	—	—	—	—	—	—	3.29	**0.004**
SAC	36	—	—	—	—	—	—	—	—	—	—	—	—

*p*-values are depicted in bold for better readability.

However, during the slow-processing condition, activations were mainly found within the SPL. Frontal channels were only significantly different from zero in the moderate- to high-speed conditions (see Table 2).

**Fig. 1:**
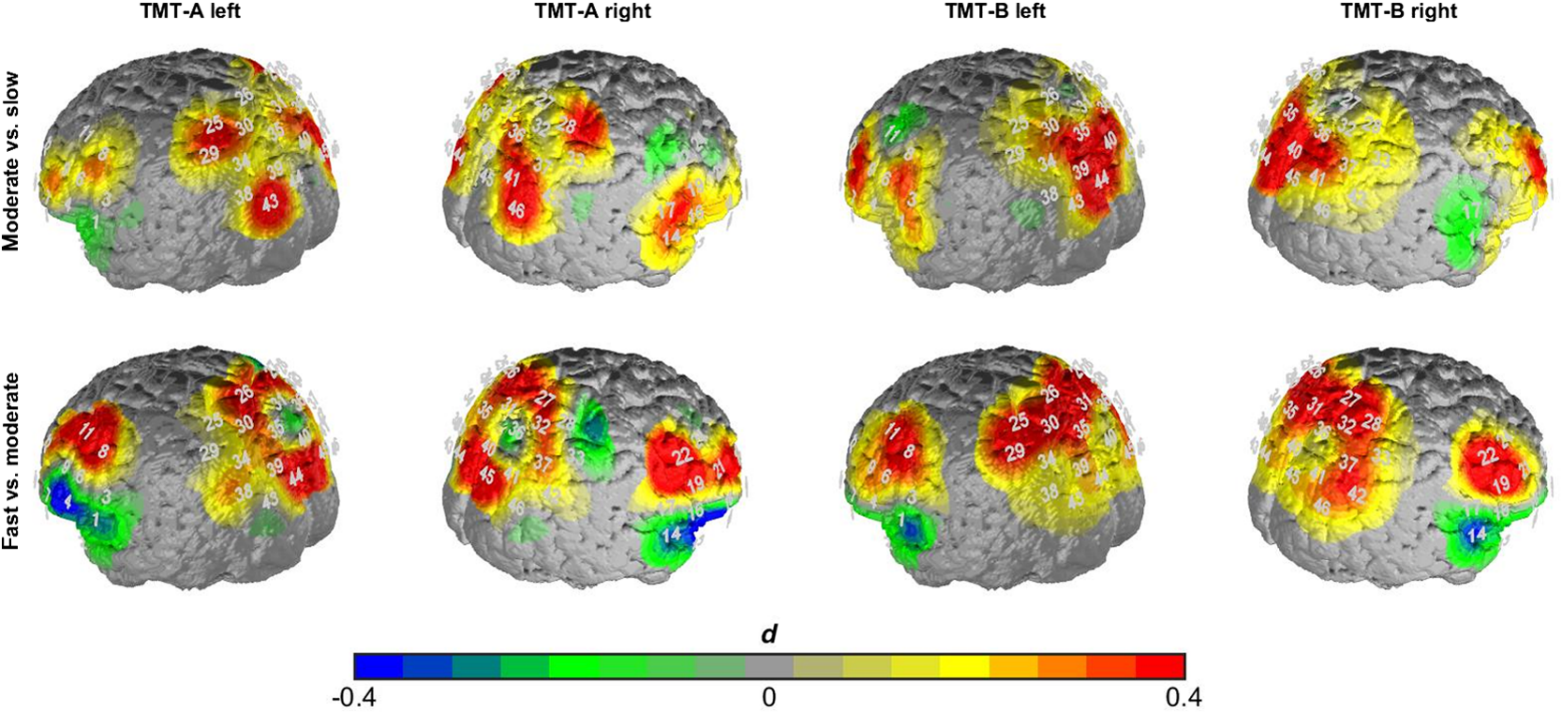
Contrast of the speed conditions in TMT-A and TMT-B. The upper row depicts the moderate- versus slow-speed contrast, and the lower row depicts the fast-versus moderate-speed contrast. Differences are shown in effect size Cohen’s d.

**Fig. 2:**
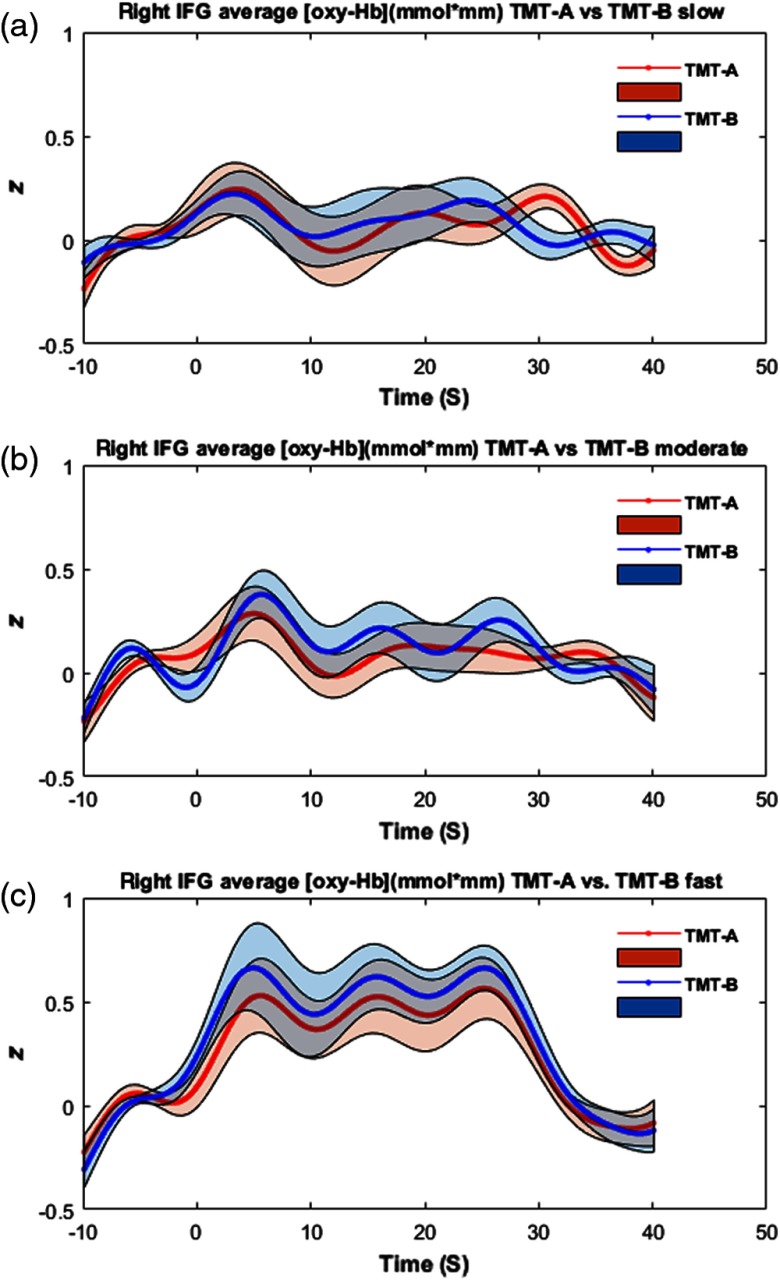
Hemodynamic responses during TMT-A (red) and TMT-B (blue) in the three speed conditions: (a) slow, (b) moderate, and (c) fast in the right inferior prefrontal cortex. Values are given in z-standardized scores of activation. Shaded areas indicate ±1 standard error of the mean.

Within the analysis of the fNIRS data, we observed main effects for processing speed in the left IFG [$F_{\left(2,38\right)}=4.98$, p<0.05, $\eta^{2}=0.21$), left DLPFC [$F_{\left(2,38\right)}=3.68$, p<0.05, $\eta^{2}=0.16$], right IFG [$F_{\left(2,38\right)}=7.25$, p<0.01, $\eta^{2}=0.28$], and SPL [$F_{\left(2,38\right)}=5.62$, p<0.01, $\eta^{2}=0.23$] as indicated by a two (TMT) by three (speed) repeated measurement ANOVA. We found no significant interaction of TMT and processing speed or a significant main effect of TMT. As indicated by post-hoc analysis, all significant main effects of speed were characterized by linear increases in the hemodynamic response from slow to fast conditions: left IFG [$F_{\left(1,19\right)}=10.32$, p<0.01, $\eta^{2}=0.35$], left DLPFC [$F_{\left(1,19\right)}=7.87$, p<0.01, $\eta^{2}=0.29$], right IFG [$F_{\left(1,19\right)}=11.54$, p<0.01, $\eta^{2}=0.37$], SPL [$F_{\left(1,19\right)}=11.38$, p<0.01, $\eta^{2}=0.37$] (see Figs. 1 and 2).

### 3.3 Processing Speed Corrected Functional Near-Infrared Spectroscopy Data

**Fig. 3:**
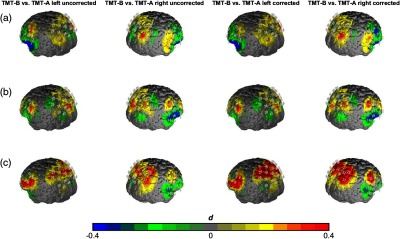
Contrast of the TMT-B versus TMT-A in the three speed conditions: (a) slow, (b) moderate, and (c) fast. The left columns depict the uncorrected $O_{2}\mathrm{Hb}$ measures and the right columns depict the ratio-corrected measures. Differences are shown in effect size Cohen’s d.

**Fig. 4:**
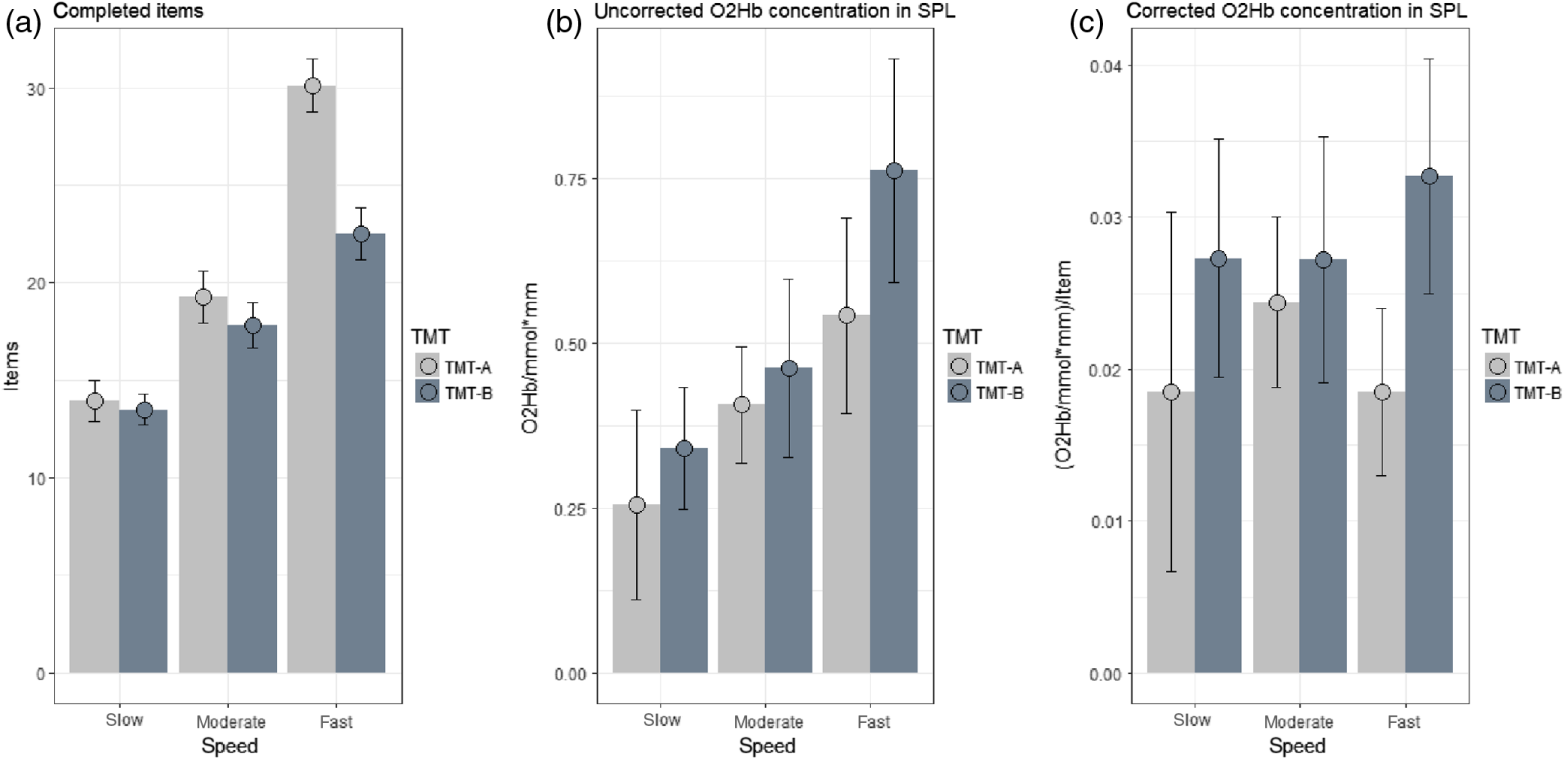
Effects of the speed condition and the TMT condition on (a) completed items, (b) uncorrected $O_{2}\mathrm{Hb}$ concentration in the SPL, and (c) corrected $O_{2}\mathrm{Hb}$ concentration in the SPL. Confidence intervals indicate 2 deviations of the standard error of the mean.

Analyses of corrected fNIRS data revealed similar results between the regression and ratio approach. In both analyses, a comparable high main effect of TMT was observed in the SPL [regression approach: $F_{\left(1,19\right)}=4.34$, p<0.05, $\eta^{2}=0.18$, ratio approach: $F_{\left(1,19\right)}=4.41$, p<0.05, $\eta^{2}=0.19$], with higher hemodynamic responses during the TMT-B in comparison to TMT-A (see Figs. 3 and 4). Note that although only the main effect of TMT was significant, in Fig. 2, a descriptive trend can be observed for higher TMT-B versus TMT-A effects in the fast-speed condition than in moderate- and slow-speed conditions.

## 4 Discussion

This study aimed to investigate the effects of processing speed (slow versus moderate versus fast) and task complexity (TMT-A versus TMT-B) on hemodynamic changes as assessed with fNIRS in a block design. Our results showed that subjects were able to perform both tasks at the instructed speed levels, as indicated by a main effect of the factor processing speed. However, the usual behavioral effect of a reduced number of solved items during the TMT-B in comparison to TMT-A was only observed in the fast-speed condition. Notably, this effect would be assumed, as differences in task complexity should be observed when subjects perform as fast as possible. With respect to hemodynamic responses, we found high effects for the factor of speed in areas associated with cognitive control. Significant increases in the hemodynamic response were found in the bilateral IFG, the left DLPFC, and the SPL. Interestingly, no significant main effects were found for the factor of TMT, although trends were observed for higher activation during TMT-B. When controlled for the number of processed items by a regression or ratio approach, we observed increased activity during the TMT-B in comparison to TMT-A in the SPL.

Our results are in line with previous investigations that sought to investigate the effects of the TMT in time-controlled experiments.^8^^,^^9^ In such experiments, usually no or only weak effects are found for the TMT contrast. From our results, we would assume that this is partly due to the lack of control for number of processed items. In the original neuropsychological measurement, the TMT is item-controlled, and time for test completion is used as a dependent variable. Adapted to neurophysiological measurements, an item-controlled version would need to use tailored windows for averaging to account for individually different working times between subjects. However, in some investigations, it may be desired to use a time-controlled version. Based on the results of this study, we would warn investigators that a time-controlled setting without accounting for the number of processed items might obscure the effects of the TMT contrast. In fact, in our investigation, the experimental effects of processing speed—as induced by different instructions—by far outnumbered the differential effects of the TMT. This becomes especially important in investigations in elderly populations since elderly might compensate impairments during the TMT-B by further slowing down speed. In future investigations, it will be interesting to investigate in how far compensation leads to higher blood oxygenation per item. Furthermore, as during TMT-A more items are completed, higher hemodynamic responses might be present due to higher motor activity at least in some areas.

The experimental manipulation of processing speed led to high increases in activation within areas associated with cognitive control, namely bilateral IFG, left DLPFC, and SPL. Increased activity within these areas during the TMT is not surprising since the TMT is thought to involve cognitive functions associated with these areas and previous fNIRS investigations of the TMT also found activity here.^8^^–^^10^^,^^14^ Both tasks—TMT-A and TMT-B—require visuospatial search function, motor speed, planning and attentional control. As depicted by the tests against zero, during slow-processing speed, only superior parietal areas showed significant hemodynamic responses as compared with baseline. As processing speed became faster in moderate and fast conditions, prefrontal areas became active. This effect might reflect the higher effort due to elevated processing speed. Indeed, this interpretation is in line with previous investigations of our group; higher activity was observed in the cognitive control network when subjects had to perform a math tasks under time pressure as compared with a task performance without time pressure.^4^^,^^5^ Of note, during the typical TMT instruction, subjects are asked to work as fast as possible and as accurately as possible, which is comparable to our high-speed condition.

Interestingly, we observed significant differences between the TMT versions when the dependent variable was corrected for the number of processed items. In line with this, previous studies used different correction methods. For example, Sankoh et al.^27^ found significant effects between the TMT versions in an fMRI study, in which wait times were tailored to control for processed items. In our results, differences between TMT-A and TMT-B were only observed in the SPL. Interestingly, lesion studies suggest that damage to the SPL is associated with impairments in tasks that involve the manipulation of material in working memory.^28^ The TMT-B, which requires task-switching and information updating in working memory during task completion, might be characterized by an increase in this cognitive function in comparison to TMT-A. Further evidence of the role of the SPL in task switching comes from functional MRI studies. For example,^29^ observed in a task-switching version of the VFT that switching conditions were characterized by increases in SPL activity in comparison to nonswitching conditions.

In this study, we compared two different correction approaches to account for the number of processed items. Both approaches—a regression and ratio approach—yielded similar results. As most studies of the TMT do not include different speed conditions (which would allow for intrasubject correction by regression), we suggest using the simple ratio approach in such investigations as an additional dependent variable to check for differences when accounting for processing speed.

Despite these important results, some limitations and considerations have to be outlined. First, the fNIRS method only allows to measure the upper part of the cortex and spatial resolution is limited. Therefore, subcortical areas could not be measured and small areas of activation might have been missed. However, as the cognitive control network is in large parts located in cortical areas, fNIRS captures most of these areas. Second, although fNIRS is rather robust in terms of movement artifacts, the TMT induces movement and arousal artifacts. Especially, artifacts from teeth clenching, which is prominent in high arousal, induces high amplitudes of inverted U-shaped artifacts in the temporal muscles that have to be corrected before further processing of the data. If not, these artifacts might be mistakenly interpreted as strong hemodynamic responses. Therefore, we used strong correction methods by reducing data variance through deletion of PCA components and an additional PCA-based kernel filter. These correction methods, however, might induce negative activation, which was not observed in this study. Third, we chose a design in which subjects were instructed to use self-imposed speed levels. From this instruction, one might argue that the instruction itself might induce some kind of mental effort since subjects had to reduce their best performance speed willingly. However, we suggest that such mental effort should lead to higher activity. Since we observed reduced hemodynamic responses within the slower-speed conditions, this factor might be negligible. In fact, we argue that the effort of the subjects increases as they had to perform faster. Although it is the aim of the used correction methods to correct for the influence of processed items, it is important to bear in mind that such a correction will also result—to some extend—in a correction for mental effort, as both constructs are associated. However, as long as subjects perform with equal effort in TMT-A and TMT-B versions (e.g., during fast speed—when performance is at the edge of skill), the correction methods will just result in a correction for processed items for the comparison of these two conditions.

Further, the study at hand aimed to investigate the effects of speed and complexity on the hemodynamic response in block designs. We only investigated a rather small sample size as we conducted a proof-of-principle study. As computed with G-Power (3.0), with the sample size of 20 subjects, effects up to $\eta^{2}=0.10$ were detectable between the TMT conditions (α=0.05, 1−β=0.80, 2 measurements, r=0.50). As we observed descriptive trends for higher hemodynamic responses during TMT-B as compared with TMT-A in the uncorrected data, it might be possible that we would have found such an effect without correction in a larger sample. However, it was not the intention of this investigation to raise any doubt on this point, but to show that the within-subject correction for completed items might yield a promising scale for the investigation of block-design hemodynamic data. It is important to bear in mind that the study at hand is no critique of block-design measurements per se, but a reminder that time-controlled settings may leave the potential confounder of performed items.

## 5 Conclusions

To the knowledge of the authors, this is the first study that investigated the effects of processing speed on activation within the cognitive control network during the TMT in a within-subject design. We observed high effects for processing speed that outnumbered the effect of the TMT contrast. In conclusion, sensitivity of the TMT contrast might be increased by implementing some sort of correction method, as during TMT-A more items are processed than during TMT-B in time-controlled block designs. The suggested methods in this article might just be a first step in search of an optimal correction, and future research might yield better approaches than the simple ratio-based correction. However, the proposed method is a simple to use correction method that might be used in any block design investigation and paradigm, in which experimental conditions differ with respect to the number of processed items.
